# LncRNA MIR205HG regulates melanomagenesis via the miR-299-3p/VEGFA axis

**DOI:** 10.18632/aging.202450

**Published:** 2021-02-01

**Authors:** Jinlan Guo, Quan Gan, Caibin Gan, Xiaoning Zhang, Xinping Ma, Mingliang Dong

**Affiliations:** 1 Xinxiang Central Hospital, Xinxiang 453000, Henan, China

**Keywords:** melanoma, MIR205HG, miR-299-3p, VEGFA

## Abstract

In this study, we investigated the role of lncRNA MIR205HG in melanomagenesis. Quantitative real-time PCR (qRT-PCR) analysis showed that MIR205HG levels were significantly upregulated in melanoma cell lines compared to normal human melanocytes. Similarly, MIR205HG levels were significantly higher melanoma tissues than adjacent normal skin tissues (n=30). CCK-8 and flow cytometry assays showed that MIR205HG knockdown significantly decreased the viability of melanoma cells. Dual luciferase reporter and RNA pull-down assays confirmed that MIR205HG directly binds to microRNA (miR)-299-3p. Targetscan analysis and dual luciferase reporter assays showed that miR-299-3p directly binds to the 3’UTR of VEGFA mRNA. Wound healing and transwell invasion assays showed that MIR205HG knockdown decreased *in vitro* migration and invasiveness of melanoma cells, and these effects were reversed by treatment with miR-299-3p inhibitor. MIR205HG-silenced melanoma cells showed increased miR-299-3p expression and lower levels of both VEGFA mRNA and protein. Tumor volumes were significantly smaller in nude mice xenografted with MIR205HG knockdown melanoma cells than the controls. These results demonstrate that MIR205HG supports melanoma growth via the miR-299-3p/VEGFA axis. This makes MIR205HG a potential therapeutic target for the treatment of melanoma.

## INTRODUCTION

Melanoma is one of the most malignant types of skin cancer with high rates of incidence worldwide [1]. Metastatic melanoma is highly resistant to chemotherapy and the survival rate of patients with advanced metastatic melanoma is only 15% [2, 3]. Therefore, there is an urgent need to identify new therapeutic targets for melanoma.

Long non-coding RNAs (lncRNAs) are a class of non-protein coding transcripts that are >200 nucleotides in length and play critical roles in several physiological processes including metabolism as well as and the development and functioning of the cardiovascular and nervous systems [4]. Moreover, lncRNAs regulate the growth and progression of multiple malignant tumors [5–7]. LncRNAs such as AFAP1-AS1, MIR4435-2HG, and LUADT1 have been implicated in the regulation of melanoma growth and progression [8–10]. LncRNA MIR205HG plays a critical role in lung squamous cell carcinoma, prostate cancer, and cervical cancer [11–13] Liu et al showed that MIR205HG expression correlated with the prognosis of melanoma patients [14]. However, the biological function of MIR205HG in melanoma has not been fully investigated.

A class of small non-coding RNAs called microRNAs (miRNAs) regulate tumor growth and progression by directly targeting specific mRNAs and downregulating the expression of critical tumor suppressor and oncogenic proteins [15, 16]. Metastatic melanoma is regulated by several miRNAs [17, 18]. MiR-299-3p is a tumor suppressor miRNA that inhibits progression of prostate cancer by modulating androgen receptor and VEGFA signaling pathways [19]. MiR-299-3p also inhibits progression and metastasis of pancreatic cancer by inhibiting the Notch1 signaling pathway via TUG1 [20]. However, the role of miR-299-3p in melanoma is unclear. Vascular endothelial growth factor A (VEGFA) plays an important role in the growth, progression, and angiogenesis in several cancers including melanomas [21–23]. Wang et al demonstrated that miR-299-3p suppressed the proliferation and invasion of human colon carcinoma cells by targeting VEGFA transcripts and inhibiting VEGFA protein expression [24]. However, the relationship between miR-299-3p and VEGFA has not been established in melanoma. In this study, we investigated the role of MIR205HG/miR-299-3p/VEGFA axis in melanoma growth and progression.

## RESULTS

### Knockdown of MIR205HG significantly reduces proliferation of melanoma cells

**Figure 1:**
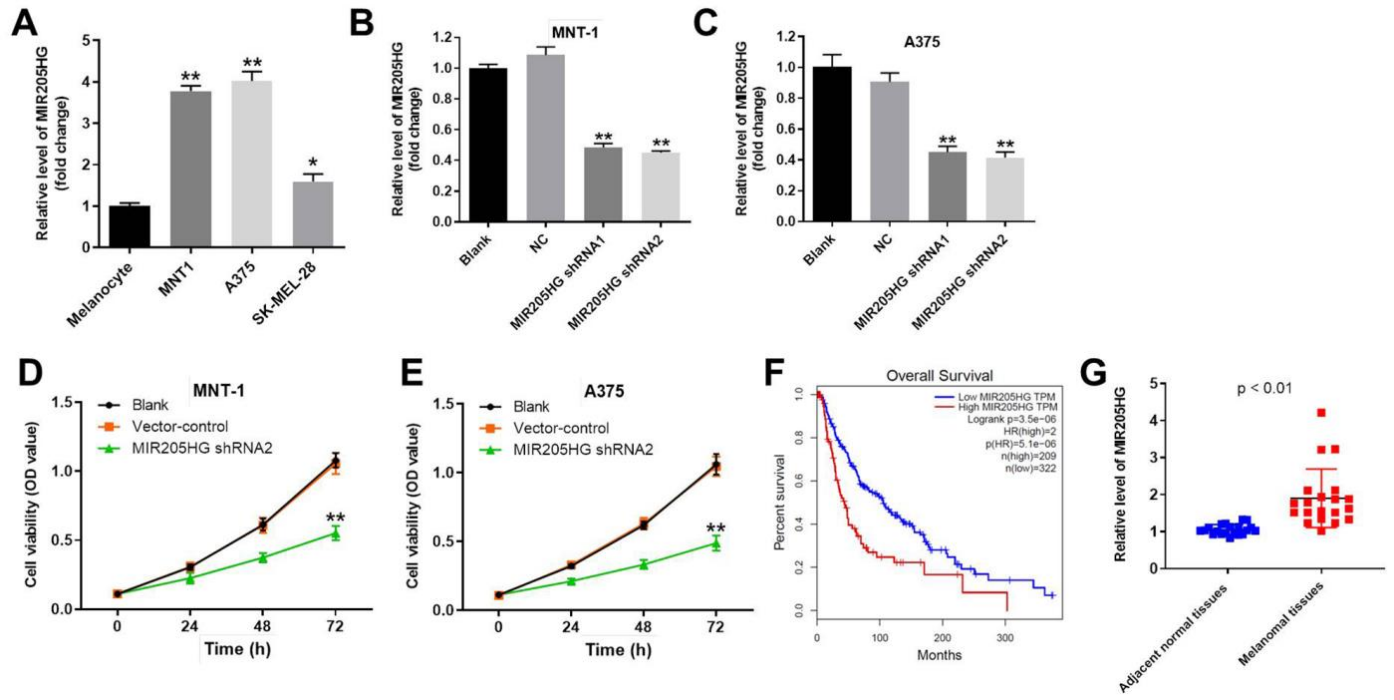
**Knockdown of MIR205HG significantly decreased the viability of melanoma cells.** (**A**) QRT-PCR analysis shows the expression of MIR205HG in melanocytes, A375, MNT-1, and SK-MEL-28 cells. (**B**, **C**) QRT-PCR analysis shows the expression of MIR205HG in (**B**) MNT-1 and (**C**) A375 cells transfected with MIR205HG shRNA1, shRNA2 or sh-NC for 24 h. (**D**, **E**) CCK-8 assay analysis results show the viability of blank, vector-control, and MIR205HG shRNA2-transfected MNT-1 and A375 cells for 0, 24, 48 or 72 h. (**F**) TCGA database analysis shows the correlation between MIR205HG expression and survival rates of melanoma patients. The analysis included the data from 531 patients with melanoma. Among the patients with melanoma, 209 patients had high expression of MIR205HG, while the others had low level of MIR205HG. (**G**) QRT-PCR analysis shows the expression of MIR205HG in paired melanoma and adjacent normal skin tissues (n=30). Note: All experiments were performed at least thrice independently. ^*^P<0.05 and ^**^P<0.01 vs. control.

Quantitative real-time PCR (qRT-PCR) analysis showed that MIR205HG levels were significantly upregulated in melanoma cancer cells (A375, MNT-1 and SK-MEL-28 cells) compared to the normal human melanocytes (Figure 1A**)**. Next, qRT-PCR analysis showed that MIR205HG levels were significantly reduced in the MIR205HG-shRNA1 and MIR205HG-shRNA2-transfected A375 and MNT-1 cells compared to their corresponding controls (Figure 1B, 1C). MIR205HG knockdown was higher in the melanoma cells with MIR205HG-shRNA2 compared to MIR205HG-shRNA1. Hence, we selected MIR205HG shRNA2 for further experiments. CCK-8 assay results showed that knockdown of MIR205HG in A375 and MNT-1 cells significantly reduced cell viability compared to the corresponding controls (Figure 1D, 1E). Furthermore, TCGA database analysis showed that high expression of MIR205HG was closely associated with lower survival rate of patients with melanoma (Figure 1F). Besides, QRT-PCR analysis showed that MIR205HG was significantly upregulated in melanoma tissues compared to the adjacent normal skin tissues (n=30; Figure 1G). Taken together, these results demonstrate that knockdown of MIR205HG significantly suppressed proliferation of melanoma cells.

### MIR205HG directly binds to miR-299-3p in melanoma cells

**Figure 2:**
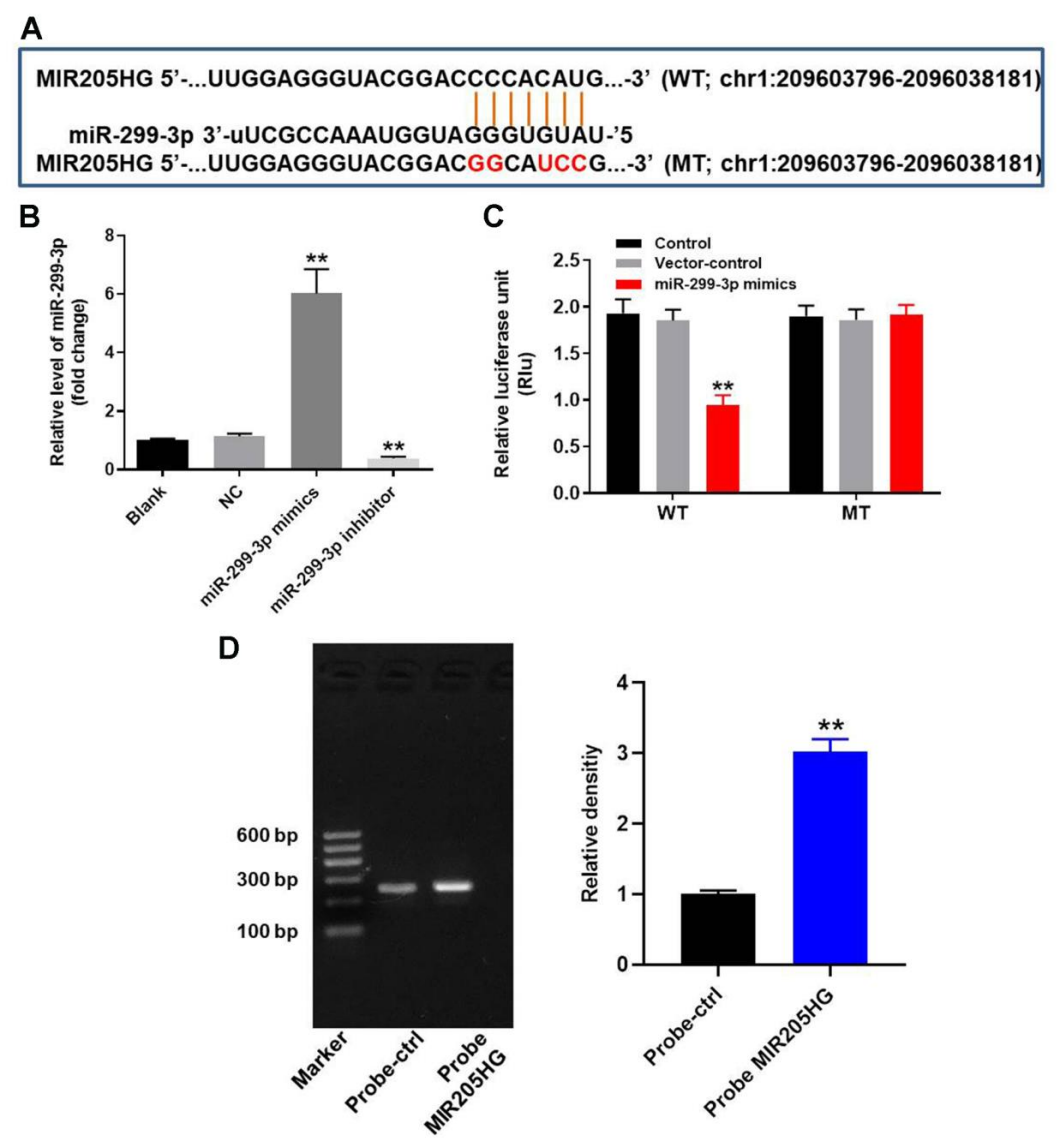
**MIR205HG directly binds to miR-299-3p.** (**A**) The predicted target binding site for miR-299-3p in the 3'UTR of lncRNA MIR205HG based on the miRDB and starBase database analysis. (**B**) Dual luciferase reporter assay results show the luciferase activity in A375 cells co-transfected with plasmid containing wild-type (WT) or mutant (MT) MIR205HG 3′-UTR and miR-299-3p. (**C**) QRT-PCR analysis shows miR-299-3p in A375 cells transfected with miR-299-3p mimics or inhibitor for 24 h and the corresponding control A375 cells. (**D**) RNA pulldown assay results show the miR-299-3p levels associated with the biotinylated MIR205HG and control probes. Note: All experiments were performed thrice. ^**^P<0.01 vs. control.

We explored the miRDB (http://www.mirdb.org/) and starBase (https://web.archive.org/web/20110222111721/http://starbase.sysu.edu.cn/) database, and then identified miR-299-3p as the most potential target miRNA of MIR205HG (Figure 2A). In addition, miR-299-3p has been reported to play a key role in progression of malignant tumor [20]. Therefore, miR-299-3p was selected from starBase and miRDB. Furthermore, dual luciferase reporter assay results confirmed that miR-299-3p was the downstream target miRNA of MIR205HG (Figure 2B). QRT-PCR analysis showed that miR-299-3p levels were significantly higher in A375 cells transfected with miR-299-3p mimics and significantly reduced in those transfected with the miR-299-3p inhibitor (Figure 2C). RNA pull-down assay showed significant enrichment of miR-299-3p with the biotinylated MIR205HG probe compared to the corresponding control (Figure 2D). Overall, these results confirmed that miR-299-3p was directly bound by MIR205HG.

### Silencing of MIR205HG induces apoptosis of melanoma cells

**Figure 3:**
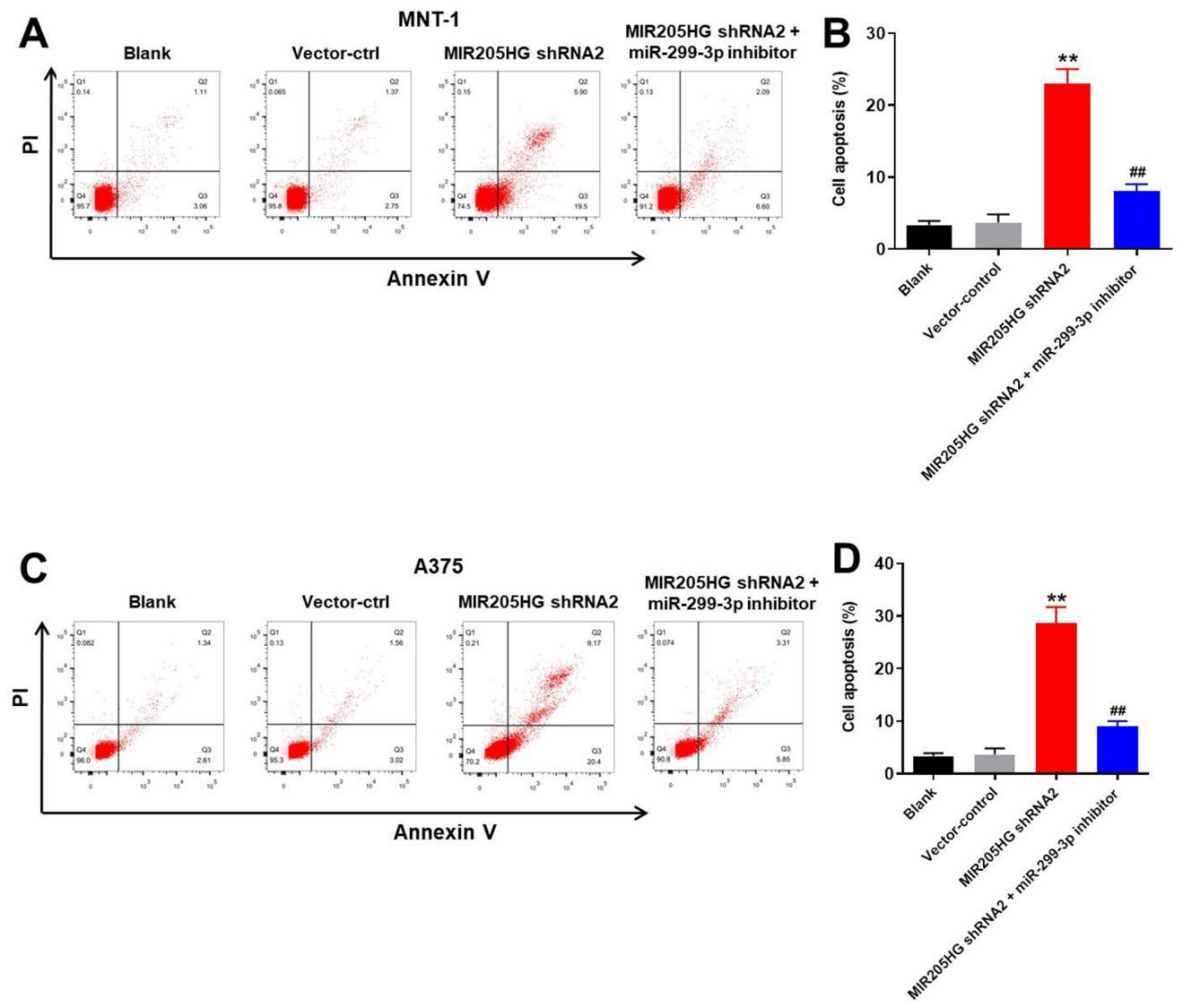
**Silencing of MIR205HG promotes apoptosis in melanoma cells.** Representative FACS plots show Annexin-V FITC (X-axis) and propidium iodide (PI; Y-axis) stained (**A**, **B**) control and MIR205HG shRNA2-transfected A375 cells and (**C**, **D**) control and MIR205HG shRNA2-transfected MNT-1 cells. The apoptotic rate was calculated based on the percentage of Annexin-V^+^ PI^+^ and Annexin-V^+^ PI^-^ cells in each group. All experiments were performed thrice. ^**^P<0.01 compared to the control; ^##^P<0.01 vs. MIR205HG shRNA2-transfected cells.

Flow cytometry analysis showed that knockdown of MIR205HG significantly increased apoptosis in the A375 and MNT-1 cells compared to the corresponding controls (Figure 3A–3D). However, treatment with the miR-299-3p inhibitor reduced apoptotic rates in the MIR205HG-knockdown A375 and MNT-1 cells (Figure 3A–3D). This suggested that MIR205HG silencing increased apoptosis of melanoma cells via miR-299-3p. We chose MIR205HG-shRNA2-transfected A375 cells for further experiments because they were more sensitive than the MIR205HG-shRNA2-transfected MNT-1 cells.

### MIR205HG silencing inhibits migration and invasion of melanoma cells via miR-299-3p

**Figure 4:**
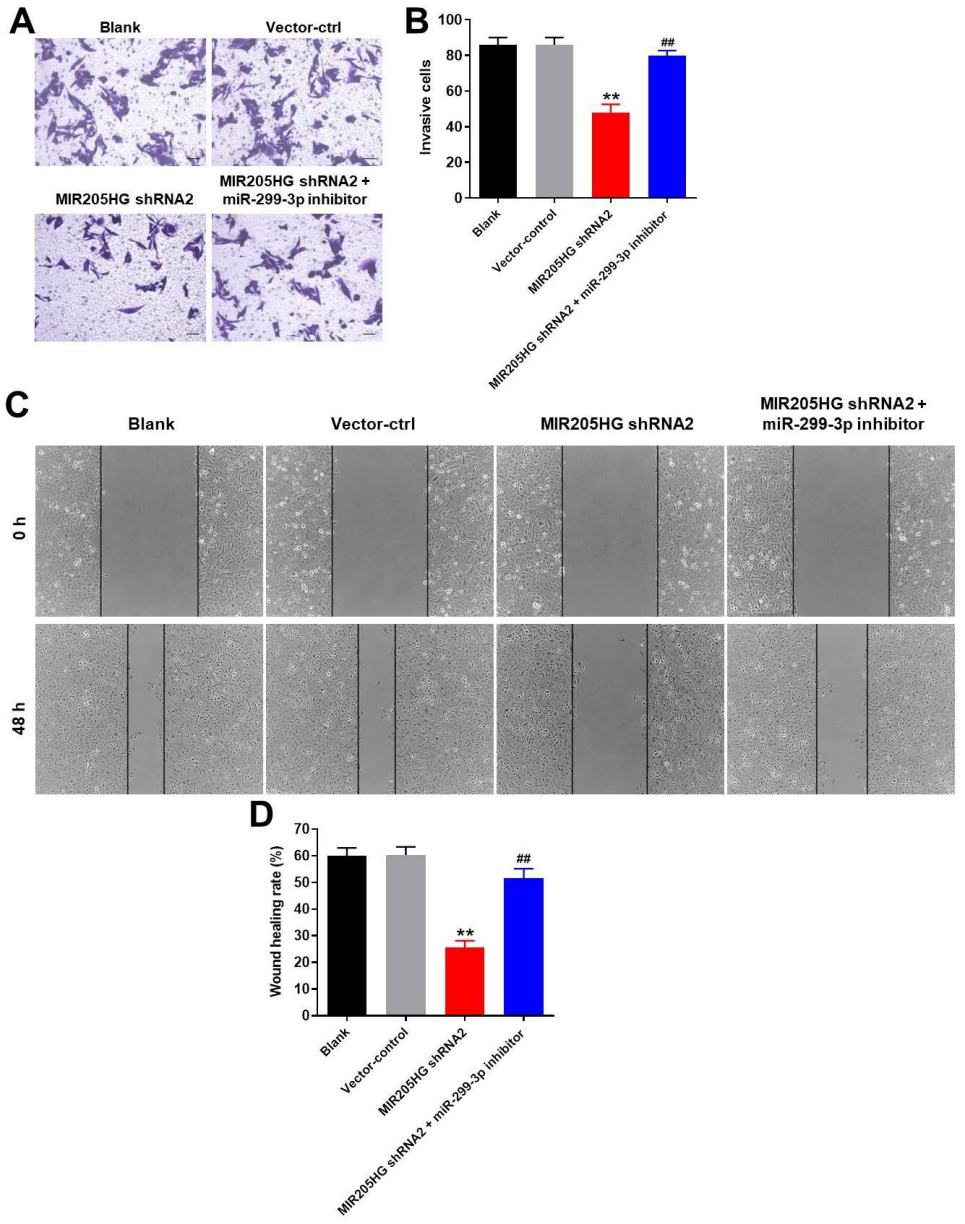
**MIR205HG knockdown inhibits migration and invasion of melanoma cells.** (**A**, **B**) Transwell assay results show the invasiveness of A375 cells transfected with MIR205HG shRNA2 or MIR205HG shRNA2 + miR-299-3p inhibitor. (**C**, **D**) Wound healing assay results show the migration ability of A375 cells transfected with MIR205HG shRNA2 or MIR205HG shRNA2 + miR-299-3p inhibitor. All experiments were performed thrice. ^**^P<0.01 vs. control; ^##^P<0.01 vs. MIR205HG shRNA2.

Next, we analyzed the effects of MIR205HG knockdown on the invasiveness and migration of A375 melanoma cells using the transwell and wound healing assays, respectively. MIR205HG knockdown significantly reduced the invasiveness of A375 cells, but these effects were partially rescued by treatment with the miR-299-3p inhibitor (Figure 4A, 4B). Furthermore, MIR205HG knockdown significantly reduced the migration of A375 cells, but, these effects were significantly reversed by treatment with the miR-299-3p inhibitor (Figure 4C, 4D). Taken together, these data demonstrate that MIR205HG silencing inhibits migration and invasion of melanoma cells via miR-299-3p.

### MiR-299-3p directly binds to 3’UTR of VEGFA in melanoma cells

**Figure 5:**
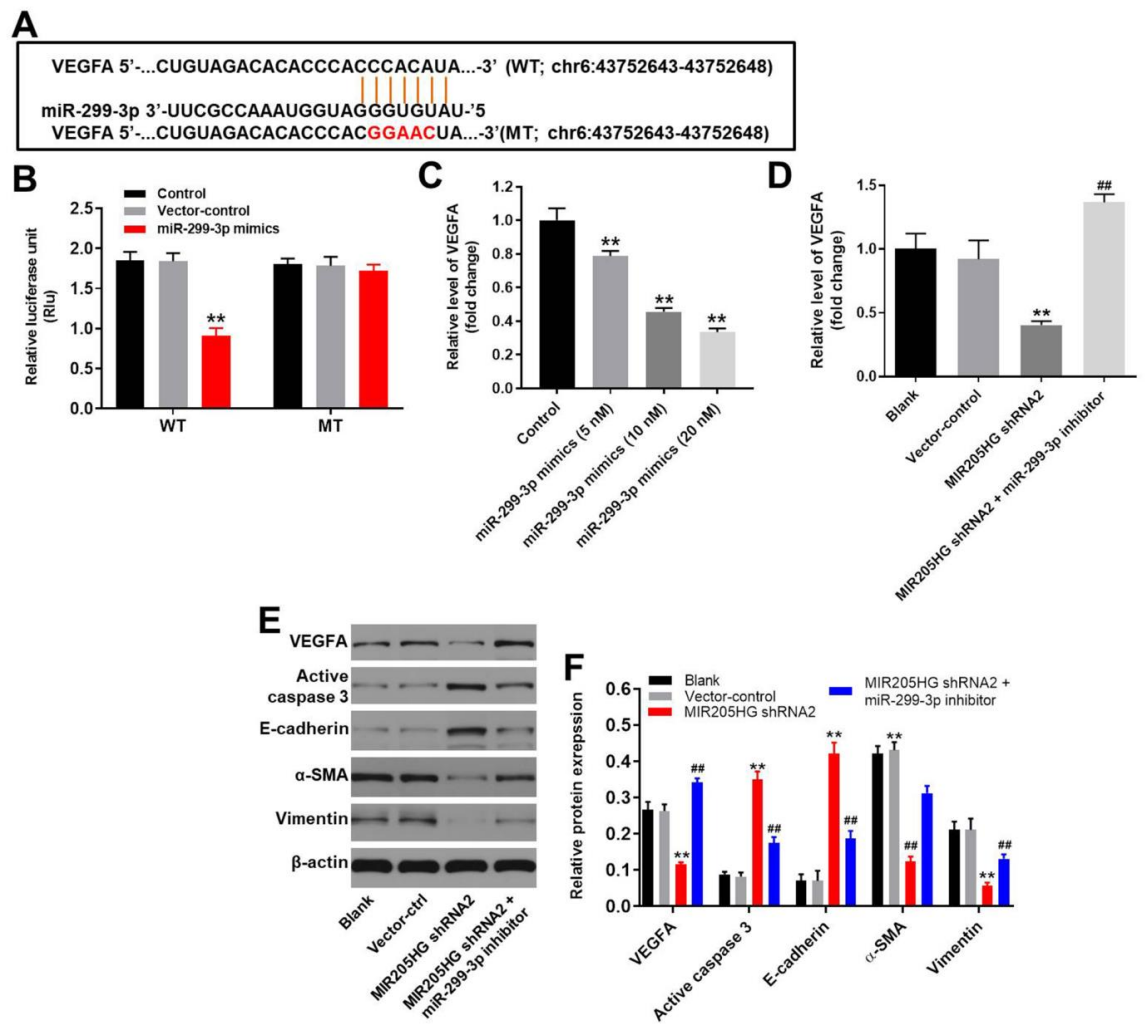
**VEGFA is the direct target of miR-299-3p.** (**A**) The predicted binding site for miR-299-3p in the 3'UTR of the VEGFA transcript between nucleotides 359-366. (**B**) Dual luciferase reporter assay results show the luciferase activity in A375 cells co-transfected with the plasmid containing wild-type (WT) or mutant (MT) VEGFA 3′-UTR and miR-299-3p. (**C**) QRT-PCR analysis shows the expression levels of VEGFA mRNA in control, 5 nM miR-299-3p mimics-transfected, 10 nM miR-299-3p mimics-transfected and 20 nM miR-299-3p mimics-transfected A375 cells. (**D**) QRT-PCR analysis shows the expression levels of VEGFA mRNA in control, MIR205HG shRNA2-transfected, MIR205HG shRNA2 plus miR-299-3p inhibitor transfected A375 cells. (**E**, **F**) Representative western blots show the levels of VEGFA, cleaved caspase3, E-cadherin, α-SMA, and Vimentin proteins in control, MIR205HG shRNA2-transfected, MIR205HG shRNA2 plus miR-299-3p inhibitor transfected A375 cells. The relative expression of these proteins was estimated using β-actin as the loading control. Note: All experiments were performed thrice. ^**^P<0.01 vs. control; ^##^P<0.01 vs. MIR205HG knockdown A375 cells.

We analyzed the targetscan (http://www.targetscan.org/vert_71/) and miRDB (http://www.mirdb.org/) database, and then identified 3’-UTR of VEGFA as a potential target of miR-299-3p (Figure 5A). Since VEGFA is involved in tumorigenesis of melanoma [21, 22], it was selected for further analysis. Furthermore, we confirmed that VEGFA was a direct target of miR-299-3p using the dual luciferase report assay (Figure 5B). In addition, miR-299-3p mimics significantly inhibited the expression of VEGFA in melanoma cells in a dose-dependent manner (Figure 5C). QRT-PCR analysis showed that the expression of VEGFA mRNA was significantly downregulated in MIR205HG shRNA2-transfected A375 cells compared to the corresponding controls, but VEGFA mRNA expression was restored partially by treatment with the miR-299-3p inhibitor (Figure 5D). Western blot analysis showed that cleaved caspase-3 and E-cadherin levels were significantly upregulated in MIR205HG-silenced A375 cells compared to the corresponding controls, but were significantly reduced in the MIR205HG-silenced A375 cells treated with the miR-299-3p inhibitor (Figure 5E, 5F). In contrast, MIR205HG shRNA2-transfected A375 cells showed reduced levels of VEGFA, α-SMA and Vimentin proteins, but these effects were reversed by treatment with the miR-299-3p inhibitor (Figure 5E, 5F). These results demonstrate that MIR205HG regulates melanoma progression via the miR-299-3p/VEGFA axis.

### Downregulation of MIR205HG or miR-299-3p agomir significantly suppressed the growth of melanoma *in vivo*

**Figure 6:**
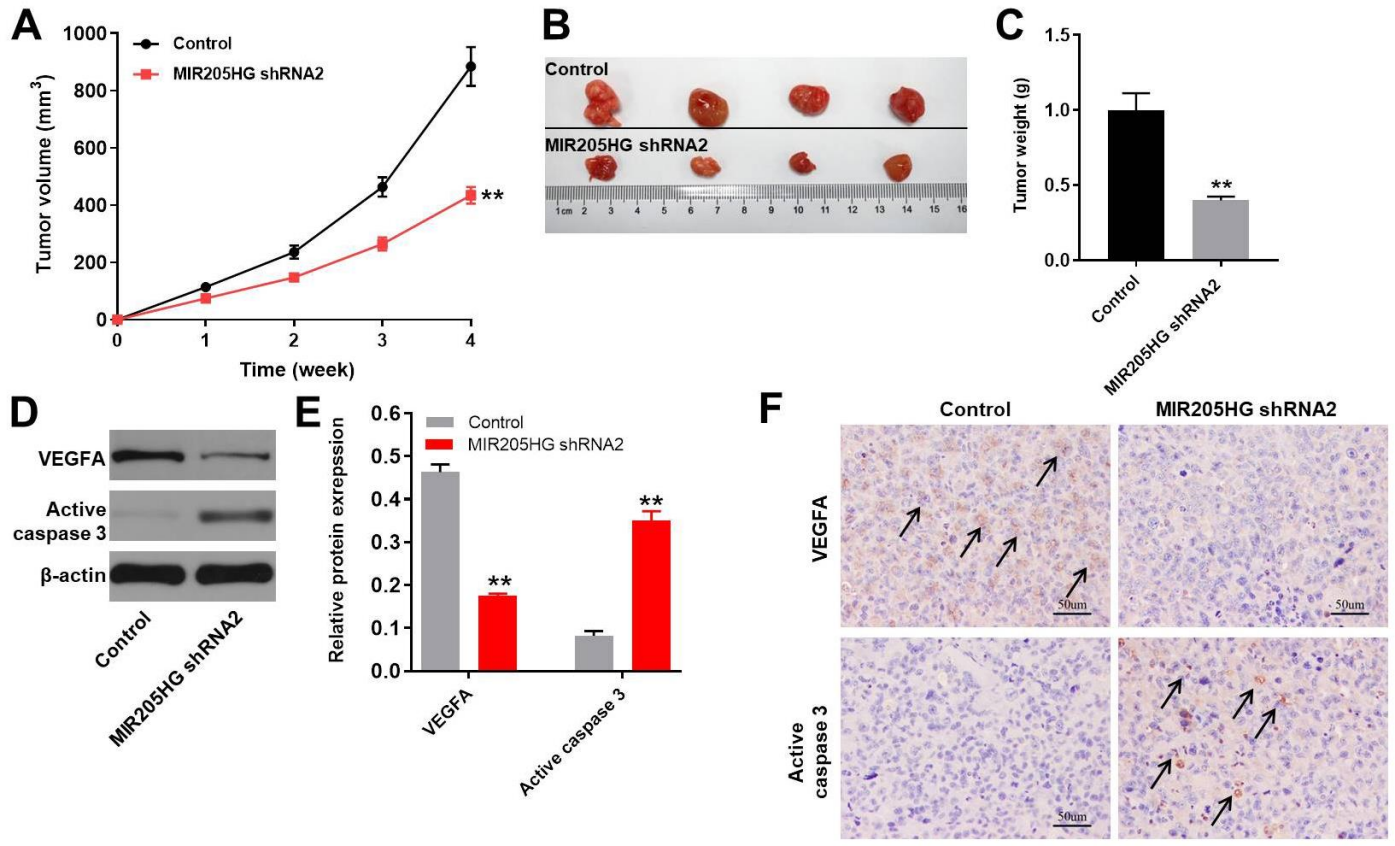
***In vivo* growth of melanoma is inhibited by MIR205HG silencing.** (**A**) Tumor volumes in nude mice subcutaneously injected with control and MIR205HG shRNA2-transfected A375 cells. Tumor volumes were measured weekly. (**B**) Representative images show the xenograft tumors in nude mice at 4 weeks after subcutaneously injecting control or MIR205HG shRNA2-transfected A375 cells. (**C**) Tumor weights in nude mice subcutaneously injected with control and MIR205HG shRNA2-transfected A375 cells (n=4 per group). (**D**, **E**) Western blot analysis shows the expression levels of VEGFA and cleaved caspase3 in xenograft tumor tissues harvested from nude mice subcutaneously injected with control and MIR205HG shRNA2-transfected A375 cells. The relative protein expression levels were quantified by normalizing to endogenous β-actin as the loading control. All experiments were performed thrice. (**F**) The expressions of active caspase 3 and VEGFA in tumor tissues of mice were detected by IHC staining. Black arrows indicate the positive cells after IHC staining. ^**^P<0.01 vs. control.

Finally, we analyzed the role of MIR205HG in melanoma by generating the xenograft melanoma model mice. We observed that tumor volumes (Figure 6A), tumor sizes (Figure 6B), and tumor weights (Figure 6C) were significantly reduced in nude mice xenografted with MIR205HG-silenced A375 cells compared to the controls**.** Moreover, VEGFA protein levels were significantly downregulated (Figure 6D, 6E) and cleaved caspase-3 levels were significantly increased (Figure 6D, 6E) in tumor tissues derived from MIR205HG-silenced A375 cells compared to the controls. Additionally, the data of IHC staining indicated that knockdown of MIR205HG notably inhibited the expression of VEGFA but increased the level of active caspase 3 in tumor tissues of mice (Figure 6F). On the other hand, miR-299-3p agomir significantly decreased the tumor size and weight in mice, while downregulation of miR-299-3p notably promoted the tumor growth of melanoma in mice (Supplementary Figure 1A–1C). Taken together, these results demonstrate that MIR205HG silencing or miR-299-3p agomir significantly inhibits the *in vivo* growth of melanoma.

## DISCUSSION

Previous reports have shown that lncRNAs play an important role in melanoma [25, 26]. In the present study, we demonstrated that MIR205HG levels were significantly upregulated in the human melanoma cells. Liu et al showed that MIR205HG expression levels were closely associated with the prognosis of melanoma [14]. Our data is consistent with this finding and suggests that MIR205HG is a potential prognostic biomarker in melanoma. A previous study reported that MIR205HG inhibits progression of cervical cancer by interacting with SRSF1 and modulating KRT17 expression [13]. This demonstrates contrasting roles for MIR205HG in different tumor types. Meng et al demonstrated that MIR205HG modulated the progression of atherogenesis by sponging miR-205-5p [30].

Next, we explored the mechanism by which MIR205HG regulated *in vitro* and *in vivo* melanoma growth and progression. We identified miR-299-3p as the downstream target miRNA of MIR205HG. MiRNAs are highly conserved small non-coding RNAs that regulate multiple biological functions by suppressing the expression of their target proteins at the post-transcriptional level by binding to the 3’UTRs of the mRNAs [15, 27]. Previous studies demonstrate that miR-299-3p plays a tumor-suppressor function in multiple malignancies [20, 28, 29]. Our study also demonstrates that miR-299-3p inhibits melanoma growth and progression.

Several studies have shown that miRNAs exert their function by downregulating the expression of their target genes by directly binding to specific miRNA-recognition sequences in the 3’UTR region of the specific target mRNAs [31, 32]. We demonstrated that VEGFA was a direct target of miR-299-3p in the melanoma cells. VEGFA plays a key role in tumor progression by promoting angiogenesis in the tumor tissues [33–36]. Our study showed that MIR205HG enhanced melanoma growth and progression by targeting the miR-299-3p/VEGFA axis. MicroRNA-299-3p inhibits the proliferation and invasion of colon cancer cells by targeting VEGFA [24]. VEGFA plays a key role in angiogenesis and invasion of several different cancer cell types [37, 38].

We also demonstrated that MIR205HG silencing inhibited the *in vitro* migration and invasion of melanoma cells. Previous studies have shown that epithelial-mesenchymal transition (EMT) process plays a critical role in the metastasis of various cancers [39, 40]. During tumor progression, E-cadherin levels are downregulated [40] and the levels of vimentin and α-SMA are upregulated [41, 42]. Furthermore, high VEGFA expression positively correlates with the EMT process [43, 44]. Taken together, our data demonstrates that MIR205HG knockdown inhibits melanoma growth and progression by suppressing VEGFA expression and EMT via miR-299-3p.

Our study has several limitations. Firstly, our study demonstrated that miR-299-3p was sponged by MIR205HG, but did not determine other MIR205HG-related miRNAs. Moreover, we only focused on the role of the VEGFA/EMT axis and did not investigate the involvement of other pathways involved in melanoma progression. Therefore, further investigations are required to unravel the mechanistic details underlying the oncogenic role of MIR205HG in melanoma.

In summary, our study demonstrates that silencing MIR205HG suppresses melanoma growth and progression by inhibiting the VEGFA expression and EMT via miR-299-3p. Thus, MIR205HG is a potential therapeutic target for melanoma.

## MATERIALS AND METHODS

### Melanoma cell lines and cell culture

We purchased human melanocytes, melanoma cell lines (MNT-1, A375, and SK-MEL-28), and 293T cell lines from the American Type Culture Collection (ATCC, Manassas, VA, USA). They were cultured in RPMI-1640 medium (ThermoFisher, Shanghai, China) with 10% fetal bovine serum (FBS) in a humidified incubator maintained at 37° C and 5% CO_2_.

### Cell transfections

We obtained lentiviral vector (pLVX-IRES-Puro) cloned with the negative control (NC) or short-hairpin RNAs against lncRNA MIR205HG (MIR205HG shRNA1 or MIR205HG shRNA2) from the Hanbio Biotechnology Co., Ltd (Shanghai, China). The negative control (NC) and MIR205HG shRNA1 or MIR205HG shRNA2 lentiviral vectors were transfected into 293T cells and the cells were incubated at 37°C for 48 h. Subsequently, we removed cell debris and cells by centrifugation at 956×g for 15 min and passed the supernatants through a 45 μm filter (Costar, Cambridge, MA, USA) to purify the lentiviral particles. We then centrifuged melanoma cells (5×10^6^/well) with the purified lentiviruses at 956×g for 15 min and then incubated the cells for 48 h in RPMI-1640 medium containing puromycin (Sigma, MA, USA) for selection. The efficiency of MIR205HG knockdown was verified by qRT-PCR.

We purchased miR-299-3p mimics, miR-299-3p inhibitor or negative control RNA (NC) from GenePharma (Shanghai, China) and transfected them into melanoma cells using Lipofectamine 2000 as previously described [45]. The efficiency of transfection was verified by qRT-PCR.

### Melanoma patient tissues

We collected 30 pairs of melanoma and adjacent normal tissues between December 2018 and December 2019 from melanoma patients at the Xinxiang Central Hospital. We also obtained clinical and pathological data of these patients as well as their written informed consent. The tissue samples were stored at -80° C. The present study was approved by the Ethics Committee of the Xinxiang Central Hospital. The information of patients has been supplemented in Table 1.

**Table 1:** The clinical information for patients with melanoma.

	**Number**	**MIR205HG High expression (n=12)**	**MIR205HG Low expression (n=8)**	**P value**
**Age**				0.6903
≥60	14	8	6	
<60	6	4	2	
**Gender**				
Male	9	5	4	0.7136
Female	11	7	4	
**Tumor size**				
≥3 cm	12	10	2	0.0091**
<3 cm	8	2	6	
**Metastasis**				
Yes	11	9	2	0.0277*
No	9	3	6	

*P<0.05, **P<0.01.

### Quantitative real time polymerase chain reaction (qRT-PCR)

Total RNA was extracted from the melanoma cell lines using TRIzol reagent (TaKaRa, Tokyo, Japan) according to the manufacturer's protocol. First-strand cDNA synthesis was performed using the PrimeScript RT reagent Kit (Takara) according to the manufacturer's protocol. Then, quantitative PCR (qPCR) was performed in an ABI7500 real-time PCR system using SYBR green(Takara). The qRT-PCR protocol was 94° C for 2 mins followed by 35 cycles of 94° C for 30 s and 55° C for 45 s.

All the qRT-PCR primers were obtained from GenePharma (Shanghai, China). These include

MIR205HG (forward): 5’-GACCGTTGTTAGCACGCCTT-3’;

MIR205HG (reverse): 5’-CACGTATCGGTCCGTGTTGG-3’;

miR-299-3p (forward): 5’-TTCCATACTGCAACGCCATACC-3’;

miR-299-3p (reverse): 5’-GCAATCCGCCCTTAGTCCAA-3’;

VEGFA (forward): 5’-GAACTTTCTGCTGTCTTGGGTG-3’;

VEGFA (reverse): 5’-GGCAGTAGCTGCGCTGATAG-3’;

U6 (forward): 5’-CGTCTTCCCAGGACCGTA-3’;

U6 (reverse): 5’-CGAATCCTGACATTAAGTCG-3’;

β-actin (forward): 5’-CCTGCGAAACACCTTGATCG-3’, and

β-actin (reverse): 5’-TCGTCATGTTCCCCACTTCG-3’.

U6 was used as reference to quantify miR-299-3p levels. β-actin was used to quantify MIR205HG levels. The relative levels of miR-299-3p and MIR205HG expression were evaluated using the 2^-*ΔΔ*CT^ method.

### The cancer genome atlas (TCGA)

The correlation between MIR205HG expression and survival rate of patients with melanoma was analyzed by TCGA. The analysis included the data from 531 patients with melanoma. Among the patients with melanoma, 209 patients had high expression of MIR205HG, while the others had low level of MIR205HG. Meanwhile, the data of TCGA was analyzed from Gene Expression Profiling Interactive Analysis (GEPIA) as previously described [46].

### CCK-8 assay

Cell counting kit-8 (CCK8, Beyotime, Shanghai, China) assay was used to analyze cell viability. We seeded 5×10^3^ MNT-1 or A375 cells per well in the blank, negative control (NC) or MIR205HG knockdown (MIR205HG shRNA2) groups in 96-well plates for 0, 24, 48 and 72 h, respectively. Subsequently, we added 10 μl CCK-8 reagent and incubated cells further for 2 h at 37° C. Then, we measured absorbance at 450 nm using a microplate reader (Thermo Fisher Scientific, Waltham, MA, USA).

### RNA pull-down

We used the Biotin RNA Labeling Mix (Roche, Basel, Switzerland) according to the manufacturer’s instructions to generate the biotinylated control and MIR205HG probes. We then transfected biotinylated-MIR205HG or control probes into the A375 cells. The RNA structure buffer (Thermo Fisher Scientific, MA, USA) was used to induce secondary structure formation in the biotin-labeled MIR205HG or control RNAs. The streptavidin beads (Thermo Fisher Scientific, Waltham, MA, USA) were washed three times with the 500 μL of RNA immunoprecipitation wash buffer (Thermo) and then incubated with the biotinylated RNAs at 4° C overnight. The overnight-incubated mixture was separated by a magnetic field to obtain streptavidin bead-RNA complexes. Then, the A375 cell lysates were incubated with the streptavidin bead-RNA complexes on a rotator at room temperature for 1 h followed by separation with a magnetic field to obtain streptavidin bead-RNA-protein complexes, which were then analyzed by qRT-PCR and western blotting.

### Western blotting

Total protein lysates from the melanoma cells were prepared by incubation with the RIPA buffer (Cell Signaling Technology, Danvers, MA, USA) on ice according to the manufacturer’s instructions. Then, equal amounts of total protein lysates were separated on a 10% SDS-PAGE. The separated proteins were transferred onto the polyvinylidene difluoride (PVDF) membranes. The membranes were then blocked with 5% BSA (Gibco, Grand Island, NY, USA) in Tris-buffered saline (TBS) containing 0.5% Tween-20 (TBST) for 60 min. The blots were then incubated overnight at 4° C with primary antibodies against VEGFA (1:1000, Abcam, CA, USA), cleaved caspase-3 (1:1000, Abcam), α-SMA (Abcam, 1:1000), E-cadherin (Abcam, 1:1000), vimentin (Abcam, 1:1000), and β-actin (Abcam, 1:1000). Then, after washing three times with 1X TBST buffer for 5 min, the membranes were incubated with horseradish peroxidase (HRP)-conjugated goat anti-rabbit IgG polyclonal secondary antibody (1:5000; Beyotime Biotechnology, Shanghai, China) at room temperature for 1 h. The blots were developed with the ECL+Plus chemoluminescence western blot system kit (Amersham, Cytiva, Shanghai, China). The density of the protein bands was measured using the ImageJ software.

### Cell apoptosis

We centrifuged 1×10^6^ MNT-1 or A375 cells per well in a 6-well plate at 1000 rpm for 5 min. Then, after removing the supernatant, we incubated the cells with 5 μl Annexin V-FITC (20 μg/ml) and 5 μl propidium (PI; 50 μg/ml) in 100 μl Annexin-V binding buffer for 15 min in the dark. The stained cells were then analyzed in a BD flow-cytometer (BD, Franklin Lake, NJ, USA) and the proportions of apoptotic cells (Annexin-V^+^ PI^-^ plus Annexin-V^+^ PI^+^) were estimated using the Fluorescence activated Cell Sorting (FACS, BD, Franklin Lake, NJ, USA) and Flowjo software (BD, Franklin Lake, NJ, USA).

### Dual luciferase reporter assay

The wild-type and mutant constructs of MIR205HG were cloned into the pmirGLO Dual-Luciferase miRNA Target Expression Vector (Promega, USA). Then, melanoma cells were co-transfected with wild-type or mutant VEGFA 3’UTR and miR-NC or miR-299-3p mimics using Lipofectamine 3000. The transfection efficacy was analyzed using pmirGLO reporter as an internal control. The luciferase activities were analyzed at 48 h using the Dual-Luciferase Reporter Assay System (Promega, USA) according to the manufacturer’s instructions. The parts, sequences and transcripts of MIR205HG were presented at https://www.ncbi.nlm.nih.gov/nuccore/NR_145433.1. The information originated from National Center for Biotechnology Information (NCBI).

### Transwell invasion assay

For the *in vitro* invasion assay, the upper chambers of the Transwell plates (Corning, New York, NY, USA) were coated with 100 μl of Matrigel (BD Biosciences, Franklin Lake, NJ, USA). Then, we seeded the melanoma cells (1×10^5^ cells) in FBS-free RPMI-1640 medium into the upper chamber, and added RPMI-1640 supplemented with 10% FBS into the lower chamber. The cells were incubated at 37° C for 24 h in a humidified incubator maintained at 5% CO_2_. Then, the cells attached to the underside of the membrane were fixed with 4% paraformaldehyde and stained with 0.5% crystal violet solution. Finally, we captured images and counted the numbers of invading cells under a light microscope.

### Wound healing assay

We seeded 5×10^3^ A375 cells into each well of a 24-well cell culture cluster plate. After the cells reached 80-90% confluence, we scraped the cell monolayer with a small pipette head. Then, after washing thrice with PBS, we added serum-free medium and cultured the cells further. We recorded the scratch widths at 0 and 48 h using an optical microscope. The experiment was repeated 3 times.

### Animal study

The *in vivo* animal experiments were performed in accordance with the National Institutes of Health (NIH) guide for the care and use of laboratory animals and the protocol was approved by the Ethics Committees of Xinxiang Central Hospital. We purchased eight 6-week old BALB/nude mice from Vital River (Beijing, China). The control, MIR205HG shRNA2, miR-299-3p agomir or miR-299-3p antagomir-transfected A375 cells (1×10^6^) were subcutaneously injected into the right flanks of the nude mice (4 mice per group) as previously described [47]. Tumor growth was measured weekly in all mice for four weeks. Tumor volume was calculated as length× (width)^2^/2 as previously described [8]. The animals were sacrificed at 4 weeks after xenografting the melanoma cells. The tumor tissues were harvested and weighed, and the expression of VEGFA and active caspase 3 in tumor tissues of mice were detected by immunohistochemistry (IHC) staining as previously reported [48].

### Statistical analysis

All experiments were performed at least thrice independently. The data are represented as means ± standard deviation (SD). The data between two groups were compared using the unpaired Student’s t-test, whereas, data between multiple groups were compared using one-way analysis of variance (ANOVA) followed by Tukey test (GraphPad Prism7). P<0.05 was considered statistically significant.

## Supplementary Material

Supplementary Figure 1
