# Supplementary material for: LncRNA MIR205HG regulates melanomagenesis via the miR-299-3p/VEGFA axis
Source: Aging (Albany NY). 2021 Feb 1;13(4):5297–311. doi: 10.18632/aging.202450 (PMC7950277; doi:10.18632/aging.202450)
Supplement: Supplementary Figure 1 [file aging-13-202450-s001.pdf]

SUPPLEMENTARY FIGURE

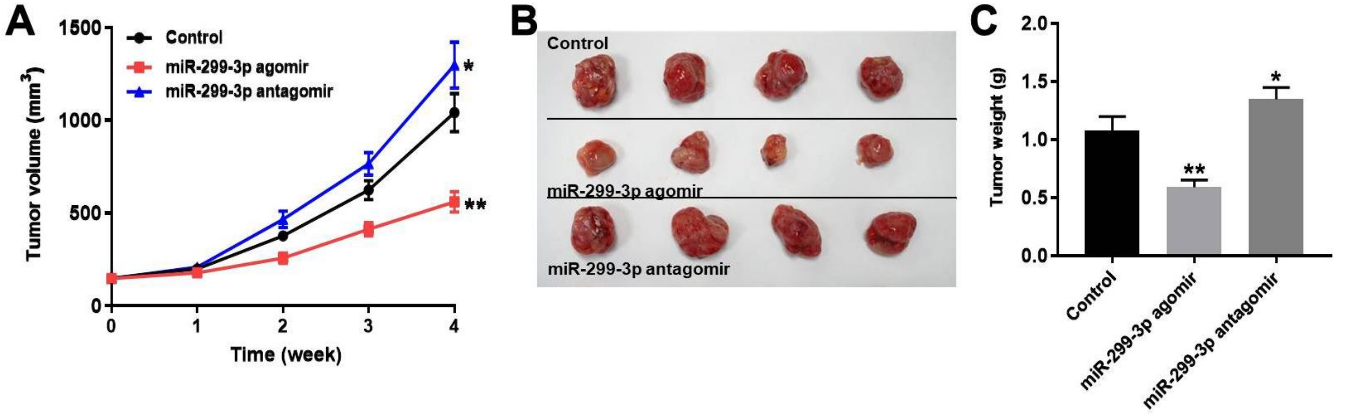

Supplementary Figure 1. MiR-299-3p agomir significantly inhibited the growth of melanoma *in vivo*. (**A**) Tumor volumes in nude mice subcutaneously injected with control, miR-299-3p agomir and miR-299-3p antagomir-transfected A375 cells. Tumor volumes were measured weekly. (**B**) Representative images show the xenograft tumors in nude mice at 4 weeks after subcutaneously injecting control, miR-299-3p agomir and miR-299-3p antagomir-transfected A375 cells. (**C**) Tumor weights in nude mice subcutaneously injected with control, miR-299-3p agomir and miR-299-3p antagomir-transfected A375 cells (n=4 per group). *P<0.05, **P<0.01 vs. control.
